# The feasibility and acceptability of collecting psychosocial outcome measures embedded within a precision medicine trial for childhood cancer

**DOI:** 10.1002/cam4.7339

**Published:** 2024-06-19

**Authors:** Eden G. Robertson, Kate Hetherington, Rebecca Daly, Mark W. Donoghoe, Nicholas Handelsman, David S. Ziegler, Claire E. Wakefield

**Affiliations:** ^1^ Discipline of Paediatrics and Child Health, School of Clinical Medicine, UNSW Medicine & Health UNSW Sydney Kensington New South Wales Australia; ^2^ Behavioural Sciences Unit, Kids Cancer Centre Sydney Children's Hospital Sydney New South Wales Australia; ^3^ Kids Cancer Centre Sydney Children's Hospital Sydney New South Wales Australia; ^4^ Stats Central, Mark Wainwright Analytical Centre UNSW Sydney Kensington New South Wales Australia; ^5^ Children's Cancer Institute UNSW Sydney Sydney New South Wales Australia

**Keywords:** acceptability, clinical trial, feasibility, oncology, outcome measures, paediatric, psychosocial

## Abstract

**Background:**

Patient‐reported outcomes measures (PROMs) are increasingly being collected within cancer clinical trials, yet limited literature on the feasibility and acceptability of doing so.

**Methods:**

We collected parent‐proxy and adolescent (≥12 years old) PROMs through a longitudinal, psychosocial sub‐study (‘PRISM‐Impact’) embedded in a precision medicine trial for children with poor prognosis cancer (‘PRISM’). We report on feasibility (response, participation, and attrition rates; follow‐up and responding to elevated distress) and acceptability (parents’ perceived benefit/burden of participation; and impact on decision to participate in PRISM) of PRISM‐Impact.

**Results:**

Over the reporting period, 462 families were eligible for PRISM‐Impact. Family and adolescent response rates were 53% and 45%, respectively. Parents whose child had relapsed were more likely to participate in PRISM‐Impact than parents whose child had not (*p* < 0.001). Parent and adolescent attrition rates were 30% and 56% respectively. We conducted 478 calls for intake and to follow‐up on missing questionnaires, and 122 calls to respond to elevated distress. Parents reported wanting to participate in PRISM‐Impact for altruistic reasons and because they valued psychosocial research. Parents reported little‐to‐no burden and some benefit from participating in PRISM‐Impact, with little change in ratings overtime. Most parents felt that participating in PRISM‐Impact did not impact their desire to participate in PRISM (72%), with some feeling more eager to participate (19%).

**Conclusions:**

PRISM‐Impact response rates were comparable to other psycho‐oncology studies, despite the poor prognosis population. Integration of PROMs within a paediatric oncology trial is acceptable to parents, and may provide a more comprehensive assessment of the impact of trial participation.

## 1 INTRODUCTION

Patient‐reported outcome measures (PROMs) empower patients to provide direct feedback on healthcare interventions. In adult care, PROMs facilitate person‐centred care, ultimately improving the quality of service provision, quality‐of‐life, symptom control, and reducing mortality.^1^ In paediatrics, PROMs via patients and proxies may provide useful feedback on the child's wellbeing and support family decision‐making.^2^, ^3^, ^4^

Patients' and caregivers' preferences are critical in decision‐making situations where treatment options have equal likelihood of cure, but differ in patient quality‐of‐life outcomes.^5^ Collecting PROMs within clinical trials may provide a more holistic understanding of the impact of new drugs/treatments.^5^ In Australia and New Zealand, ~45% of all registered trials include at least one patient‐reported outcome endpoint.^6^ With the adoption of any new treatment approach such as precision medicine, having a comprehensive understanding of the impact on patients and caregivers, and their perspectives, is necessary to ensure successful wide‐scale implementation.

Researchers and clinicians need more support to collect PROMs within cancer trials.^7^, ^8^, ^9^ Several guidelines provide recommendations on collection of PROMs within a clinical trial protocol^10^ and how to report PROMs for cancer clinical research.^11^ However, there is a lack of guidelines to support researchers regarding how to collect these data ‘in‐trial’.^7^, ^12^ To our knowledge, there is also minimal research evaluating the acceptability and/or feasibility of collecting PROMs within childhood cancer clinical trials, particularly in trials recruiting patients with a poor prognosis and trials adopting a precision medicine approach. PRecISion Medicine for Children with Cancer’ (PRISM) is an Australia‐wide multi‐site clinical trial that aims to test the feasibility of providing precision medicine for paediatric and adolescent patients with poor prognosis malignancies.^13^, ^14^ Specifically, PRISM aims to provide personalised treatment recommendations for children, based on the unique characteristics of their tumour and germline. Following consent to PRISM and collection of a tumour sample, scientists conduct an array of molecular tumour profiling and drug testing. The results from these tests are discussed at a multidisciplinary tumour board and curated in a report for the treating oncologist, who communicates findings to the family. If a potentially beneficial treatment is identified through this process, it is at the discretion of the treating oncologist and patient/family as to whether there is any change in disease management.

Running alongside PRISM (recruitment completed: December 2023), ‘PRISM‐Impact’ is a prospective, longitudinal psychosocial study which aims to collect PROMs and parent‐proxy measures to better understand families' attitudes toward, and the impact of, PRISM.^15^, ^16^ PRISM‐Impact collects data from families 2–4 weeks post PRISM enrolment (T0), 2–8 weeks after the delivery of PRISM results and any treatment recommendation(s) to families (T1), and then annually up to 5 years post PRISM enrolment (T2–T4). PRISM‐Impact questionnaires, anticipated to take less than 30 min to complete each, included a mix of purpose‐designed and validated measures; (see Appendix S1). Questionnaires were presented at a Grade 7.0 readability level (Flesch‐Kinkaid readability score), and deemed appropriate by consumer representatives.

In this study, we aimed to answer these research questions (RQs):

1. How feasible is it to collect PRISM‐Impact data?
2. Why do parents participate in PRISM‐Impact?
3. How acceptable do families find participating in PRISM‐Impact?

## 2 METHODS

We conducted our study in accordance with the Declaration of Helsinki and received Institutional Board Approval (HREC/17/HNE/29).

### 2.1 PRISM and PRISM‐Impact

PRecISion Medicine for Children with Cancer (PRISM) is an Australia‐wide multi‐site clinical trial that aims to test the feasibility of providing precision medicine for paediatric and adolescent patients with poor prognosis malignancies.^13^, ^14^ Following consent to PRISM and collection of a tumour sample, scientists conduct an array of molecular tumour profiling and drug testing. The results from these tests are discussed at a multidisciplinary tumour board and curated in a report for the treating oncologist, who communicates findings to the family. If a potentially beneficial treatment is identified through this process, it is at the discretion of the treating oncologist and patient/family as to whether there is any change in disease management.

Running alongside PRISM (primary data completion: December 2023), ‘PRISM‐Impact’ is a prospective, longitudinal psychosocial study which aims to collect PROMs and parent‐proxy measures to better understand families' attitudes toward, and the impact of, PRISM.^15^, ^16^ PRISM‐Impact collects data from families up to 5 years post enrolment to PRISM.

PRISM‐Impact questionnaires, anticipated to take less than 30 min to complete each, include a mix of purpose‐designed and validated measures; (see Appendix S1). Questionnaires were presented at a Grade 7.0 readability level (Flesch‐Kinkaid readability score), and deemed appropriate by consumer representatives.

### 2.2 Recruitment

Families consented to PRISM‐Impact through PRISM's consent process. Children and adolescents were eligible for PRISM if they were ≤21 years old and diagnosed with a poor prognosis malignancy (<30% chance of survival and anticipated life expectancy ≥6 weeks). All parents of patients were eligible for PRISM‐Impact, as were patients who were ≥12 years at PRISM enrolment. We excluded participants with insufficient English or presented with any mental/physical concerns that in the opinion of their treating team would interfere with their ability to participate. In this paper, we report on the data of families with a child <18 years old.

Two weeks after consenting, we telephoned their parents to conduct an intake. During intake, we explained the PRISM‐Impact study, confirmed consent for one or two parents (and adolescent, if applicable), ascertained preferences for questionnaire format, obtained contact details for a healthcare professional (for a mental health emergency) and screened for elevated distress using the Distress Thermometer.^17^ If a second parent was participating, we noted whether to send questionnaires separately.

### 2.3 Study procedures

**FIGURE 1:**
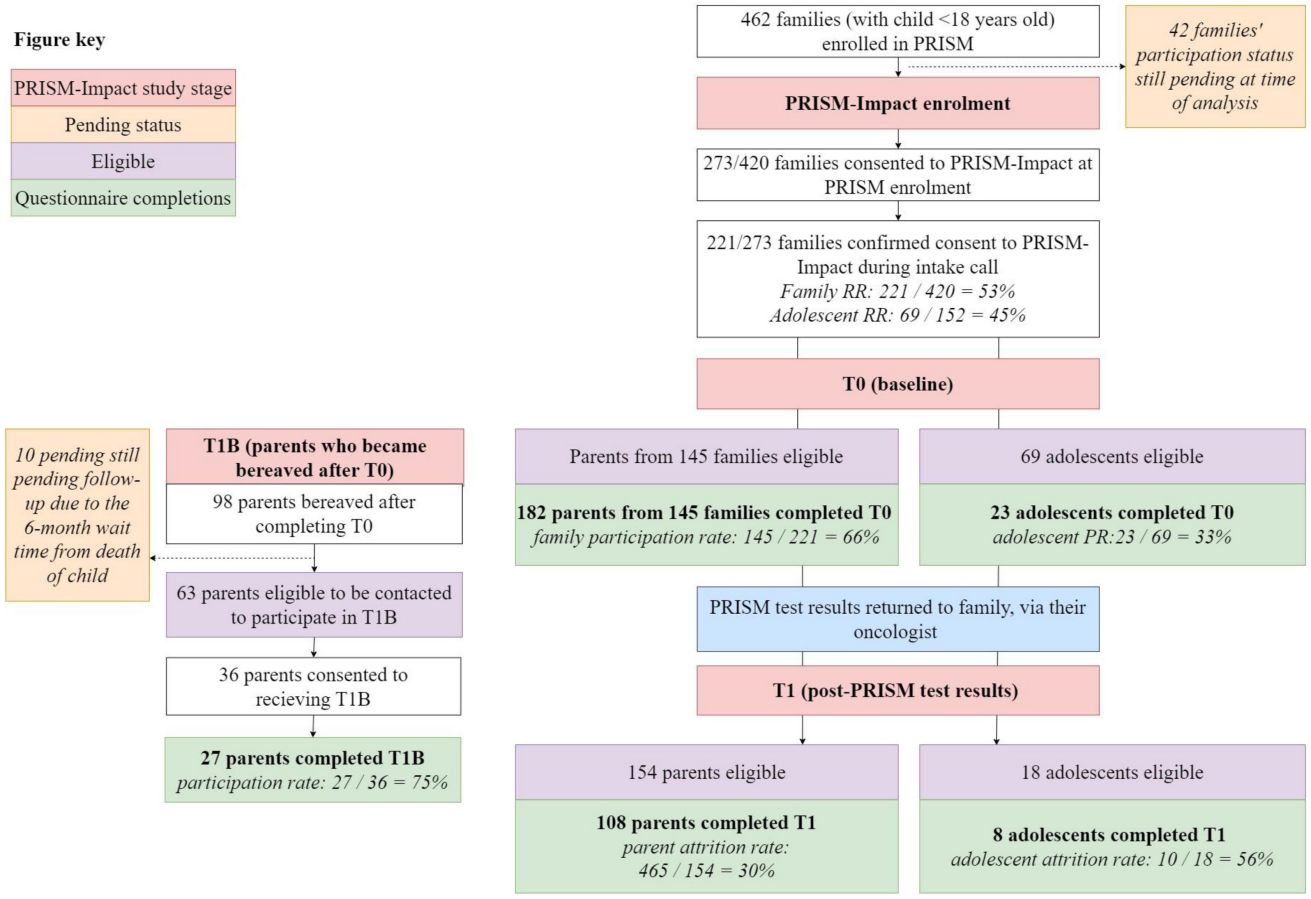
PRISM‐Impact recruitment and study flow. Eligible participants at T0 differ to those eligible for T1 or T1b due to participants revoking their consent, being considered unreachable/lost to follow‐up, and/or the PRISM database not indicating that they had received their test results.

Once participants confirmed consent, we sent their baseline questionnaire (T0: PRISM enrolment). If paper questionnaires were sent, we included a reply‐paid envelope. We conducted follow‐up calls to those who did not return their questionnaire within 2 weeks, and only contacted adolescents via their parent. We deemed participants ‘unreachable’ if they did not answer three follow‐up call attempts, or ‘maxed out’ if they answered a call and still did not return their questionnaire. For participants who completed T0, we sent their second questionnaire (T1) after the PRISM database indicated that test results had been communicated by their oncologist. We conducted follow‐up call procedures as per T0 (see Figure 1).

We excluded the parents whose child died before T0. For those whose child died after T0 and were ‘active’ (i.e., not maxed out or unreachable prior to the death of their child), we contacted the family 6 months post‐death to see whether they wanted to continue their participation via a purposely designed questionnaire (T1b). This timeframe aligns with recommendations from previous literature.^18^ We conducted follow‐up calls as per T0 and T1.

To ensure participant safety, our study psychologist contacted participants who scored ≥8 on any Emotion Thermometers Tool (ETT)^17^ domain in a survey and/or indicated ‘yes’ on desire for further help, or expressed distress during contact with the study team. For adolescents, our study psychologist first contacted their primary parent to get permission to contact their child.

### 2.4 Data collection

With expected final data completion for PRISM‐Impact in December 2028, this paper reports on data collected during the first 3 years of PRISM‐Impact (2017–2021). Specifically, this includes data collected from participants at intake, T0 (at PRISM enrolment), T1 (after return of PRISM results) and T1b (bereaved parent survey after PRISM entry), and from the PRISM and PRISM‐Impact databases (see Appendices S1 and S2).

At T0, parents shared their reason for consenting to PRISM‐Impact via an open‐ended item. At T0 and T1, parents rated whether participating in PRISM‐Impact made them feel ‘more’ or ‘less’ eager for their child to participate in PRISM, or if it had ‘no effect’. At T0, T1 and T1b, parents rated their level of burden and benefit of being involved in PRISM‐Impact from 1 (not at all) to 5 (very much).

## 3 ANALYSIS

We conducted analyses using R (v4.0.0).

### 3.1 RQ1

We calculated the following rates:

1. Family response.
2. Family participation.
3. Parent attrition.

Adolescent response, participation and attrition rates were calculated using the relevant formula above (Appendix S3).

We used logistic regression models to investigate factors associated with: (i) parents' and adolescents' decision regarding whether to participate in PRISM‐Impact, and (ii) attrition between T0 and T1 (Appendix S4). We excluded parents with missing data on any of the included variables.

We used basic descriptive statistics to report on preferred questionnaire format, time to return questionnaires, the number of participants who indicated high distress, and the time for the psychologist to make distress management calls. We calculated the number of follow‐up calls for each missing questionnaire that was eventually returned. We do not report on calls for unreturned questionnaires.

### 3.2 RQ2

Two researchers (EGR, RD) independently conducted a directed qualitative content analysis of parents' reasons to participate in PRISM‐Impact. Parents could provide more than one reason for participating in PRISM‐Impact. The researchers met to discuss any discrepancies in coding of data until consensus was reached.

### 3.3 RQ3

We used an ordinal logistic regression model to determine whether parents' perceived burden and benefit of being involved in PRISM‐Impact changed from T0 to T1. We used descriptive statistics to report whether parents perceived participating in PRISM‐Impact affected their desire to participate in PRISM.

## 4 RESULTS

See Figure 1 for an overview of participation, and Table 1 for demographics.

**TABLE 1:** Demographics of PRISM‐Impact participants at T0.

Parent demographics	Participating parents (*N* = 182)
Age, years	
Mean (SD)	41.6 (7.4)
Range	23–67
Sex, *n* (%)	
Female	114 (62.6)
Male	68 (37.4)
Highest level of education	
High school	32 (17.6)
Apprenticeship	8 (4.4)
Certificate/diploma	52 (28.6)
University undergraduate	53 (29.1)
University postgraduate	37 (20.3)
Employment status, *n* (%)^a^	
Employed full‐time	82 (45.1)
Employed part‐time or casual	48 (26.3)
Not employed	14 (7.6)
Home duties	32 (17.6)
Marital Status, *n* (%)	
Never married/never de facto	3 (1.6)
Currently married or de facto	158 (86.8)
Separated/divorced/previous de facto/widowed	21 (11.5)
Cultural background, *n* (%)^b^	
Western/European	140 (76.9)
Other	37 (20.3)
English as a first language, *n* (%)^a^	
Yes	154 (84.6)
No	27 (14.8)

Child demographics	Participating adolescents (*N* = 23)
Age, years^a^	
Mean (SD)	14.9 (2.1)
Range	12–18
Sex, *n* (%)	
Female	14 (60.9)
Male	9 (39.1)
Diagnosis *n* (%)	
Sarcoma	12 (52.2)
CNS	5 (21.7)
Leukaemia/Lymphoma	4 (17.4)
Other	2 (8.7)
Number of relapses, *n* (%)	
0	11 (47.8)
1 or more	12 (52.1)

*Note*: The age restriction is based on the child's age at the date of consenting to PRISM, while the age summarised in the table is that reported by the parent at baseline. Hence it is possible to include children aged 18 at baseline, if they had their birthday in between PRISM consent and baseline.

Abbreviations: CNS, central nervous system; SD, standard deviation.

^a^ Missing = 1.

^b^ Missing = 5.

### 4.1 RQ1: Feasibility of PRISM‐Impact

#### 4.1.1 Responses rate

Of the 462 families (child <18 years) eligible for PRISM‐Impact, 273 families opted in to PRISM‐Impact during the PRISM consent process. Of these families, 221 confirmed their consent to during their PRISM‐Impact intake call (family response rate = 53%, 221/[462–42 families pending final follow‐up]). One hundred and fifty‐two patients were 12–17 years old at the time of consent to PRISM (33%), with 69 consenting to PRISM‐Impact (adolescent response rate = 45%, 69/152).

#### 4.1.2 Participation rate

Most families (*n* = 147, 81%) opted to receive their questionnaire online. Of the 221 families who confirmed their participation at intake, 145 families had at least one member return T0 (family participation rate = 66%, 145/221). Most had one parent participate (73%, 106/145), resulting in a total of 182 parents having completed T0. Of the 69 adolescents who were consented to participate, 23 returned T0 (adolescent participation rate = 33%, 23/69).

After adjusting for all other variables in the model, there was strong evidence that parents whose child had relapsed prior to PRISM had a higher rate of completing T0 compared to those whose child had not relapsed (OR = 2.46, 95% CI: 1.53–3.96, *p* < 0.001). There was some evidence to indicate that parents' participation differed by the child's cancer type (*p* = 0.049). For example, after adjusting for child's age, site and relapse status, parents who had a child diagnosed with a sarcoma had an adjusted odds ratio of 0.76 (95% CI: 0.43, 1.35) compared to those with CNS; see Table 2. Similarly, there was some evidence that parents' participation differed by their child's treating hospital (*p* = 0.035). For example, at John Hunter Children's Hospital the family participation was 45% versus 83% at the Monash Children's Hospital. There was no evidence to indicate that parents' participation differed by child's age; or that adolescent participation rates differed by their age, cancer type, relapse status, or treating hospital (all *p* > 0.05).

**TABLE 2:** Cancer type and treating hospital by participation rates.

Child's cancer type	Adjusted OR (95%) [vs CNS]
Sarcoma	0.76 (0.43, 1.35)
Leukaemia/Lymphoma	0.40 (0.20, 0.80)
Neuroblastoma	0.63 (0.27, 1.48)
Other	0.40 (0.18, 0.89)

Treating hospital	Family participation rates^a^
Monash Children's Hospital	83%
Queensland Children's Hospital	77%
Sydney Children's Hospital	71%
Royal Children's Hospital Melbourne	65%
Royal Adelaide Hospital	64%
Perth Children's Hospital	62%
Children's Hospital at Westmead	52%
John Hunter Children's Hospital	45%

^a^ Overall participation rate was 66%; The child's cancer type is categorised based on the medical records entered by the treating team; odds ratios were not taken for treating sites due to small sample sizes and risk of Type II errors.

#### 4.1.3 Attrition

On average, the time taken to receive PRISM test results was 14.2 weeks after PRISM enrolment (SD = 5.3). Of the 182 parents who completed T0, 154 were eligible for T1 (28 parents of 24 children died before T1). Of these, 108 parents returned T1 (parent attrition = [154–108]/154 = 30%). The most common reasons recorded for attrition were that the family was maxed out or unreachable (*n* = 29) or the child became terminally ill (*n* = 5). There was no evidence to indicate an association between maintaining participation and parent sex, child's diagnosis, relapse prior PRISM, receipt of treatment recommendations, parents' baseline ETT scores or PRISM‐Impact benefit or burden ratings (all *p* > 0.05).

Of the 23 adolescents who completed T0, 18 were eligible for T1 (five adolescents died before T1). Of the eligible adolescents, eight returned T1 (attrition = [18–10]/18 = 56%). The most common reasons recorded for attrition were that the adolescent/family were maxed out or unreachable (*n* = 4) or the adolescent became terminally ill (*n* = 3).

Ninety‐eight parents became bereaved after completing T0 (including parents who became bereaved after completing T1). Of these parents, 63 were eligible to be contacted within our study period to receive T1b (20 = deemed unreachable for T1; 5 = withdrawn after completing T0 and prior to bereavement; 10 = pending follow‐up). Of these, 36 consented to receiving the T1b (37%), with 27 (75%) returning it.

#### 4.1.4 Follow‐up calls for participating individuals

The median time for the return of the T0 was 15.5 days (range: 0–115 days). Fifty percent of the families (*n* = 72) returned T0 within 2 weeks of it being sent. Most remaining families who eventually returned T0 required 1–2 follow‐up calls (72%). In total, we made 138 follow‐up calls to these families, from which 107 calls (78%) resulted in contact.

In the process of undertaking follow‐up calls for T0, we inadvertently contacted three families who informed us that their child had died. During the calls, the research team expressed their sympathy, apologised for contacting the family, and explained that a checking procedure was in place to avoid these scenarios. The research team recorded protocol deviations, informed the PRISM team of the child's status, and labelled the family as ineligible/do not contact. In response to the first two occurrences (that both occurred on a Monday, with the child's death over the weekend), the research team established a new policy to not contact families on Mondays which provided a buffer day for the PRISM database to be updated.

The median time for the return of the T1 was 23 days (range: 0–151 days). Eighteen families (17%) returned T1 within 2 weeks. Most remaining families who eventually returned T1 required 1–2 follow‐up calls (65%). In total, we made 89 follow‐up calls to these families, from which 67 calls (75%) resulted in contact.

The median time for return of T1b was 11 days after it being sent (range: 0–179 days). Two bereaved parents (12%) returned T1b within 2 weeks. Of the remaining 15 families, most required 1–2 follow‐up calls (32%). In total, we made 33 follow‐up calls to bereaved families, from which 20 calls (61%) resulted in contact.

#### 4.1.5 Responding to indications of elevated distress

At intake, we identified 23 parents as experiencing elevated distress. Thirteen (57%) consented to receive a call from the study psychologist to discuss further. The study psychologist made a total of 34 calls in response, 71% of which resulted in contact (median attempted contacts per case = 2, range: 1–6).

Throughout points of data collection, we identified 32 parents at T0, 24 parents at T1 and 5 bereaved parents at T1b as experiencing elevated distress. Of these, a total of 39 parents consented to receive a call from the study psychologist. In response, our psychologist made a total of 88 calls, 53 (60%) of which they successfully made contact (median number of attempted contacts per case = 1.5, range: 1–4).

At T0, four adolescents indicated elevated distress via their ETT ratings. No further action was required after speaking to the parents of three adolescents. For one adolescent, the study psychologist spoke directly with the adolescent at the request of the parent. There were no cases of high distress recorded for adolescents at T1.

No participants were deemed at immediate risk of serious harm to themselves or others by the study psychologist.

### 4.2 RQ2: Reason for participating in PRISM‐Impact

Altruism was the main reason parents reported that they decided to participate in PRISM‐Impact (*n* = 90/182)

*My decision to participate in PRISM‐Impact was to contribute my experiences in the hope other people in my circumstance may benefit*. —Mother, child aged 12 diagnosed with an ‘Other Neoplasm’

*If our experiences can help researchers to develop preventive or intervention strategies to help patients and families to navigate this situation, we were happy to help*. —Father, child aged 2 diagnosed with a ‘CNS tumor’

Parents described wanting to participate as they acknowledged the importance of psychosocial research and were advocates for doing more of this type of research (*n* = 22/182).

*I believe it is important to have insight into the psychosocial aspect of this significant event in our lives… I think it should be offered for all major health diseases*. —Father child aged 10 diagnosed with a ‘CNS tumor’

*I feel parents' psychological state is very overlooked and limited resources are supplied to help parents deal with mental health, so I feel it is vital studies bring light to this situation*. —Mother, child aged 1 diagnosed with a ‘sarcoma’

Other reported reasons included: participation was of no burden (*n* = 17/182), to help their child/family (*n* = 13/182), PRISM‐Impact was recommended by their medical team (*n* = 13/182) or wanting to share their perspective (*n* = 9/182).

### 4.3 RQ3: Acceptability of PRISM‐Impact

At T0, most parents (*n* = 167, 72%) indicated that participating in PRISM‐Impact had ‘no effect’ on their eagerness to participate in PRISM. However, 32 parents (19%) indicated that it made them ‘more eager’. Some reasons provided included: *input from parents/family are being respected*, *research is looking at the holistic approach, not just the medicine* and *I'd forgotten about having signed up for it until I was called to participate. It prompted me to read more into PRISM to refresh my memory and learn more about it*. Fourteen parents (8%) indicated that it made them ‘less eager’ to participate in PRISM, mainly acknowledging the time commitment/capacity at time of invitation or the negative emotion that came with responding to surveys, such as *it brings up painful feelings and thoughts*.

**FIGURE 2:**
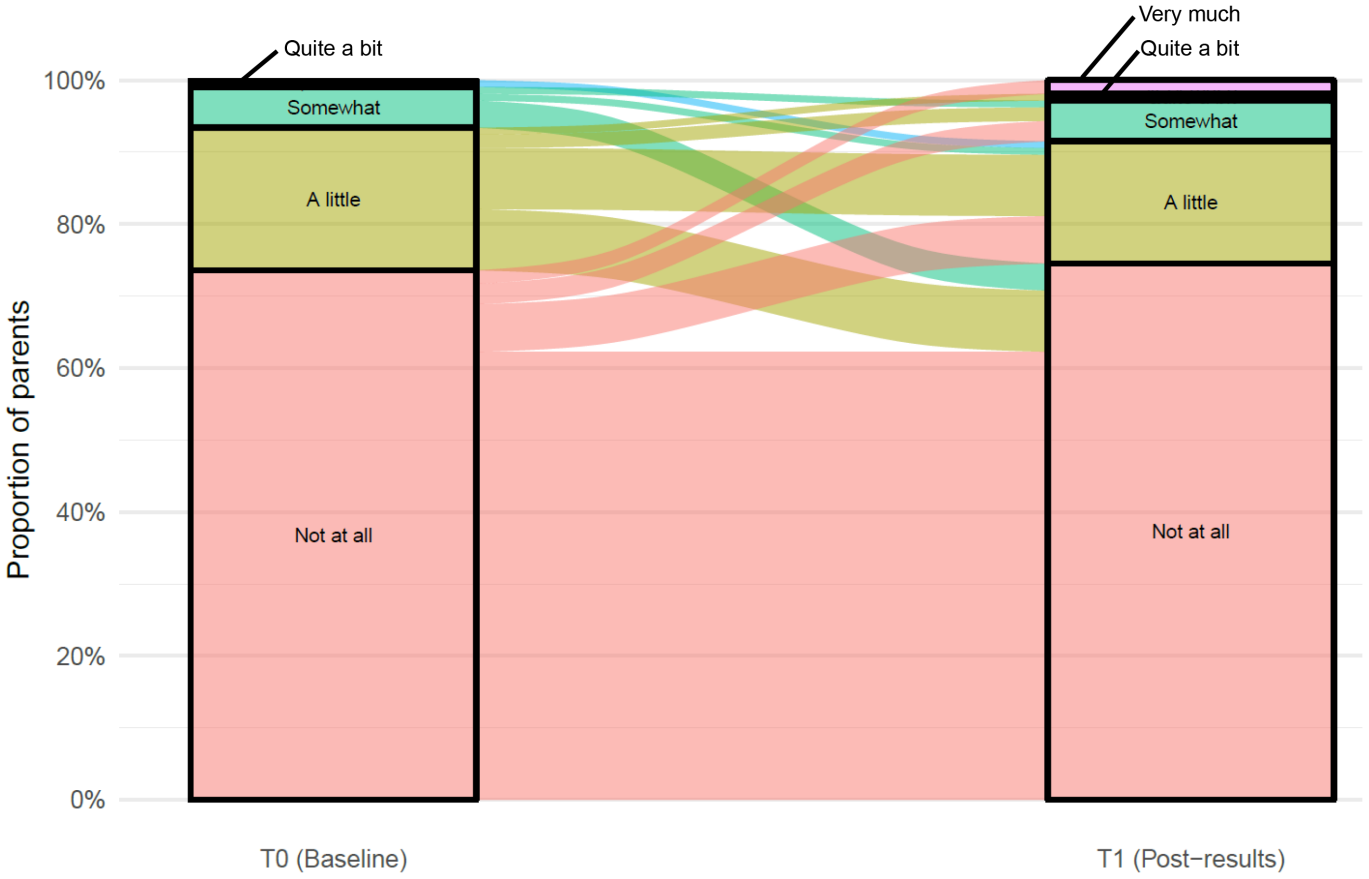
Parents' perceptions of burden in participating in PRISM‐Impact. Data only from participants who responded at both T0 and T1, excluding T1B.

**FIGURE 3:**
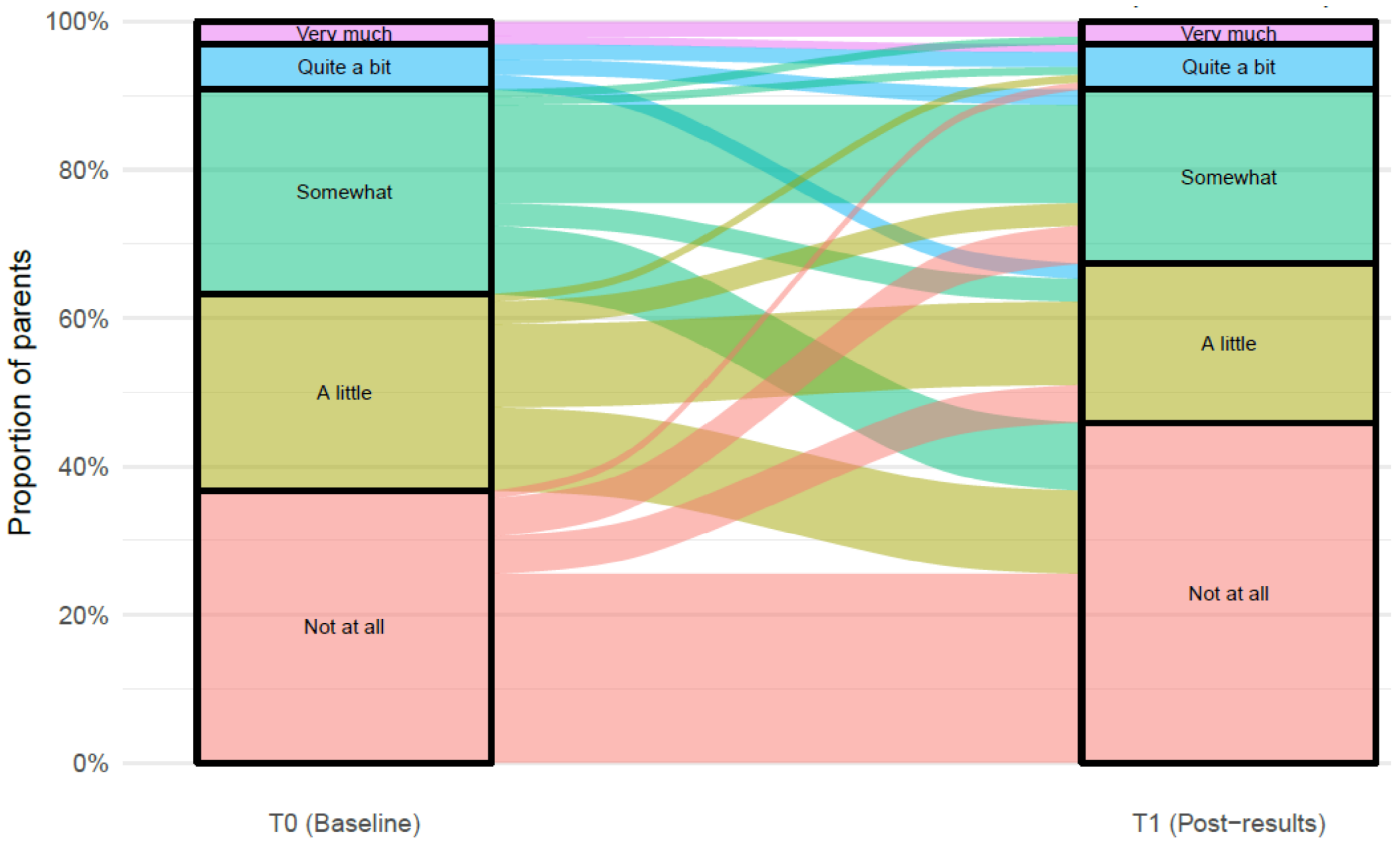
Parents' perceptions of benefit in participating in PRISM‐Impact. Data only from participants who responded at both T0 and T1, excluding T1B.

At T0 and T1, most parents reported ‘little’ or ‘no’ burden from participating in PRISM‐Impact (*n*_T0_ = 164, 94%; *n*_T1_ = 99, 92%), with about one‐third of parents reported at least ‘some’ benefit from participating (*n*_T0_ = 62, 37%; *n*_T1_ = 33, 31%). For parents who responded at both timepoints (*n* = 106), ratings of burden did not substantially change over time on average (OR = 0.98, 95% CI: 0.52–1.84, *p* = 0.946) (Figure 2), nor did ratings of benefit (OR = 0.73, 95% CI: 0.42–1.24, *p* = 0.243) (Figure 3). At T1b, most bereaved parents (*n* = 26, 96%) reported ‘little’ or ‘no’ burden from participating in PRISM‐Impact, and ‘little’ or ‘no’ benefit (*n* = 23, 88%). For those who responded at both T0 and T1b (*n* = 27), we observed a general reduction in the reported level of benefit over time (OR = 0.19, 95% CI: 0.06–0.60, *p* = 0.005).

## 5 DISCUSSION

This study reports on the feasibility and acceptability of collecting patient‐reported and parent‐proxy reported outcomes within a precision medicine trial for poor prognosis childhood cancer. We showed that while data collection takes time and specialised skills, integrating such this process within a clinical trial for precision medicine is ultimately acceptable, minimally burdensome to parents, and may even be of benefit to some. The data obtained through PRISM‐Impact will inform the national implementation of precision medicine for all children diagnosed with cancer in Australia, beyond the PRISM trial. PRISM‐Impact findings will facilitate a more holistic understanding of the impact of precision medicine (e.g., impact on quality of life,^15^ hopes and concerns^19^) and highlights areas for intervention (e.g., information provision to support understanding and decision making^16^) to improve patient experience and outcomes.

We reported an overall family participation rate of 66%, which appears lower than that reported in a 2017 systematic review of longitudinal, psychosocial studies within childhood cancer.^20^ Our response rates and attrition may be due to our poor‐prognosis cohort. Previous research conducted with parents at PRISM enrolment shows that most parents reported that their child was experiencing at least some difficulty across more than one quality of life domain,^15^ and this is likely to impact parents' decision to undertake any additional ‘tasks’ that take time away from their child.^21^ However, we found that parents whose child had relapsed prior to enrolling in PRISM were more likely to participate in PRISM‐Impact compared with parents whose child had not. It is possible that newly diagnosed families were overwhelmed with a new diagnosis; and parents at relapse are more motivated to support research given that their child will likely need experimental intervention. Further research is needed to examine these hypotheses. Most participants in our study participated in PRISM‐Impact for altruistic reasons. The benefit and lack of burden that many families reported in our study suggests that there is a level of ‘reciprocal altruism’—where parents experience benefit from ‘giving back’.^22^, ^23^ The continued involvement of bereaved parents in our study reinforces this explanation. Previous research also reiterates the benefit for parents from participating in psychosocial research at end‐of‐life and into bereavement.^24^

Recruitment and intake calls, data collection, data entry/database management, and distress follow‐up were undertaken by a dedicated, specialist team that was separate from the clinical team. The team included members with psychology expertise (e.g., a clinical psychologist/postdoctoral research fellow) and research officers (with undergraduate qualifications in social sciences or psychology). Given the complexity of the study and highly vulnerable population, a thorough study manual is used alongside the ethics‐approved protocol. The specialist team also meets weekly to discuss study progress and key challenges. Our study findings highlighted that with a dedicated, specialist team, collecting PROMs within a precision medicine trial for poor prognosis childhood cancer is feasible. Our findings are complemented by the work of Bradford et al. who estimated an average of 98 min of experienced research nurse time to recruit one participant to a paediatric psycho‐oncology research study.^25^ Signorelli et al. also estimated recruitment costs for a one‐off questionnaire with childhood cancer survivors to be ~AUD20,200 for a potential 1176 participants.^26^ Our research, combined with these previous, highlights the substantial infrastructure required to recruit, manage, and collect data. Even with rigorous protocols and trained staff, we still mistakenly contacted three families of deceased patients. This stresses the importance of having a skilled team to confidently manage difficult conversations they do not foresee.^27^ Collecting PROMs may also not be feasible for healthcare professionals given competing clinical priorities and a lack of training.^28^, ^29^ Integrating specialist researchers to collect PROMs within clinical trials may improve response rates,^30^ and the rate of meaningful, translational research. Health economic studies are required to further delineate costs and benefits of doing so.

Given the health status of patients, our primary data source was parents. It is critical that the patient voice is also captured given the reported incongruence between patient and proxy reporting in previous studies.^2^, ^3^, ^31^ Integrating proxy‐PROMs to explore psychosocial functioning is integral in providing a more comprehensive understanding of the patient's overall health. This is especially important within precision medicine where treatment‐related side effects, and impact on quality of life is largely unknown. Common to our research and previous studies is that non‐completion of PROMs is often because the patient is too ill, or perceptions that it would be too burdensome and thus participation is not offered.^30^ Our findings indicate that while some individuals experience burden from participating in PRISM‐Impact, a proportion also fund benefit from it. This included bereaved parents. Despite this, efforts to reduce questionnaire length, provide easy‐to‐read information sheets and questionnaires, and offering alternative completion formats such as face‐to‐face at the bedside, may be valuable.

### 5.1 Limitations

We reported on the response and participation rates, and follow‐up contact per family, rather than per participant, given the challenge in disentangling this data. We were limited in our ability to determine the representativeness of our data as we did not have access to extensive demographics of PRISM participants who did not partake in PRISM‐Impact, nor their reason to not participate in PRISM‐Impact. We did not collect acceptability ratings from adolescent patients.

Our sample size at T1 and T1b limited the power to conduct further analyses (e.g., analysis of change in burden ratings for bereaved parents). As T1b was sent out based on date of the child's death, changes observed may not necessary be due to bereavement. Our logistic regression models may not be ideal if missing responses are ‘missing not‐at‐random’. It is possible that the declining health of the child contributed to attrition, however this could not be well accounted for given the sample size and large confidence intervals. Further, we did not run any regressions to explore attrition and benefit/burden of PRISM‐Impact depending on questionnaire format due to sample size limitations.

### 5.2 Conclusions

Collecting PROMs requires considerable effort from a specialist research team. Participation in such studies appears of little burden to parents and may benefit some. Our findings provide guidance to future researchers aiming to collect PROMs within clinical trials, and justifies funding dedicated research staff to maximise the collection of high‐quality, longitudinal data.

## AUTHOR CONTRIBUTIONS

**Eden G. Robertson:** Conceptualization (lead); formal analysis (supporting); investigation (equal); methodology (lead); supervision (equal); validation (supporting); visualization (supporting); writing – original draft (lead); writing – review and editing (lead). **Kate Hetherington:** Conceptualization (supporting); methodology (equal); project administration (lead); supervision (lead); writing – original draft (supporting); writing – review and editing (supporting). **Rebecca Daly:** Data curation (equal); investigation (equal); project administration (supporting); validation (equal); visualization (equal); writing – original draft (supporting); writing – review and editing (supporting). **Mark W. Donoghoe:** Data curation (equal); formal analysis (lead); investigation (equal); methodology (equal); visualization (equal); writing – review and editing (supporting). **Nicholas Handelsman:** Conceptualization (supporting); data curation (supporting); methodology (supporting); project administration (supporting); validation (supporting); writing – review and editing (supporting). **David S. Ziegler:** Funding acquisition (lead); investigation (lead); methodology (supporting); writing – review and editing (supporting). **Claire E. Wakefield:** Conceptualization (equal); funding acquisition (lead); investigation (supporting); methodology (equal); supervision (supporting); writing – review and editing (supporting).

## FUNDING INFORMATION

CEW is supported by the National Health and Medical Research Council of Australia (APP2008300). KH is supported by the Cancer Institute Translational Program Grant (2021/TPG2112) as well as Luminesce Alliance and the Zero Childhood Cancer National Personalised Medicine Program for children with high‐risk cancer, a joint initiative of Children's Cancer Institute and Kids Cancer Centre, Sydney Children's Hospital, Randwick. RD and NH are supported by the Luminesce Alliance. This work was supported by Luminesce Alliance—Innovation for Children's Health. Luminesce Alliance is a not‐for‐profit cooperative joint venture between the Sydney Children's Hospitals Network, the Children's Medical Research Institute, the Children's Cancer Institute, the University of Sydney, and the University of New South Wales Sydney. It has been established with the support of the NSW Government to coordinate and integrate paediatric research. The Behavioural Sciences Unit is proudly supported by the Kids with Cancer Foundation. DSZ is supported by grants from the National Health and Medical Research Council (Synergy Grant #2019056, and Leadership Grant APP2017898) and Cancer Institute New South Wales Program Grant (TPG2037).

## CONFLICT OF INTEREST STATEMENT

DSZ reports consulting / advisory board fees from Bayer, Astra Zeneca, Accendatech, Novartis, Day One, FivePhusion, Amgen, Alexion, and Norgine and research support from Accendatech.

## ETHICS STATEMENT

We conducted our study in accordance with the Declaration of Helsinki and received Institutional Board Approval (HREC/17/HNE/29).

## CLINICAL TRIAL REGISTRATION

The ClinicalTrials.gov Identifier for the precision medicine trial, PRISM, is NCT03336931. The PRISM‐Impact study which we report data from in this study is a prospective, longitudinal mixed‐methods sub‐study that involves no intervention and thus does not require registration.

## PRECIS

Integration of patient (and proxy) reported outcome measures within a paediatric oncology precision medicine trial is acceptable to parents. High‐quality data collection may provide a more comprehensive assessment of the impact of trial participation.

## Supporting information


Appendix S1.

Appendix S2.

Appendix S3.

Appendix S4.

## Data Availability

The data that support the findings of this study are available on request from the corresponding author, EGR The data are not publicly available due to ethical restrictions.
