# Supplementary material for: The feasibility and acceptability of collecting psychosocial outcome measures embedded within a precision medicine trial for childhood cancer
Source: Cancer Med. 2024 Jun 19;13(12):e7339. doi: 10.1002/cam4.7339 (PMC11187165; doi:10.1002/cam4.7339)
Supplement: Supplementary file 1 — Appendix S1. [file CAM4-13-e7339-s001.docx]

**Appendix 1. Overview of measures included in PRISM-Impact questionnaires**

| **Measure / Domain** | **Purpose** | **Type** | **N^o^ of items** | **Parent** | | | **Adolescent** | |
| --- | --- | --- | --- | --- | --- | --- | --- | --- |
|  |  |  |  | **T0** | **T1** | **T1B** | **T0** | **T1** |
| Demographics | To capture demographics such as participants’ age and sex, child’s age, postcode | Purpose-designed | 22 | x |  |  |  |  |
| Understanding of, and satisfaction with, PRISM | To measure the extent to which participants engaged with the PRISM information sheet, reason for decision to participate in PRISM | Purpose-designed | 24 | x |  |  | x^a^ |  |
| Satisfaction with decision | To measure overall satisfaction with decision to participate in PRISM | Purpose-designed | 1 | x | x | x | x | x |
| Involvement in decision | To identify how involved the young person was in deciding whether to participate in PRISM | Purpose-designed | 1 |  |  |  | x |  |
| ‘Quality of Informed Consent’ scale | To measure level of perceived and actual understanding of the purpose of PRISM | Adapted from validated measure | 9 | x | x |  |  |  |
| Hopes and expectations from participating in PRISM | To measure hopes and expectations of participating in PRISM | Adapted from previous study | 12 | x | x | x | x^b^ | x^b^ |
| Concerns about participating in PRISM | To measure worry about participating in PRISM | Purpose-designed | 9 | x | x | x |  |  |
| Perceived impact of participating in PRISM | To measure perceived likelihood of benefits and outcomes from PRISM tests | Purpose-designed | 5 | x | x^c^ | x^c^ | x^d^ |  |
| ‘EQ-5D-5L’ | To measure quality of life measure | Validated | 6 | x | x | x | x^e^ | x^e^ |
| ‘EQ-5D-5L - parent proxy’ ^d^ | To measure quality of life of the child, from the perspective of the parent | Validated | 6 | x | x |  |  |  |
| ‘Emotion Thermometers Tool’ | To identify acute distress and need for emotional help | Validated | 5 | x | x | x | x | x |
| ‘Intolerance of Uncertainty – Short version’ | To measure the tendency of someone to consider the possibility of a negative event occurring as unacceptable, irrespective of the probability of occurrence | Validated | 12 | x |  |  |  |  |
| ‘Fear of Progression – Questionnaire – Short form – Parent’ (FoP-Q-SF/PR) | To assess the extent to which parents develop the fear of further cancer progression | Validated | 12 | x | x |  |  |  |
| Data Sharing Concerns | To measure perceptions of the risks of data sharing. | Purpose-designed | 10 | x |  | x |  |  |
| Benefit and burden of PRISM and PRISM-Impact | To measure the benefit and burden of participating in PRISM and PRISM-Impact | Purpose-designed | 8 | x | x^f^ | x^f^ | x^g^ | x^g^ |
| Information preferences from PRISM tests | To identify the types of information parents want to receive from studies like PRISM | Purpose-designed | 5 |  | x |  |  |  |
| Germline testing questions | Questions regarding whether they received any germline genetic test results from the PRISM study, and perceptions of genetic test results. | Purpose-designed | 6 |  | x |  |  |  |
| Choices regarding treatment and use of results | To determine whether any findings from the PRISM tests were considered and/or implemented. | Purpose-designed | 6 |  | x |  |  |  |
| ‘Decisional Regret scale’ | To assess level of decision regret regarding consent to PRISM | Validated | 5 |  | x | x |  |  |
| ‘Satisfaction with Decision Scale’ | To assess level of satisfaction regarding the decision to consent to PRISM | Validated | 5 |  | x | x |  |  |

**Note**. Wording of items that were used across the bereaved parents survey and adolescent surveys were adapted as necessary

^a^ only 10 questions were asked in the adolescent version

^b^ only 9 questions were asked in the adolescent version

^c^ only 1 item was asked at T1 and T1B

^d^ parents only asked if their child was aged 4 or older

^e^ ‘EQ-5D - Youth version’ was used for the adolescent version

^f^ only 7 items were asked at T1 and T1B

^g^ an additional 2 questions were asked for the adolescent version
