# Supplementary material for: The feasibility and acceptability of collecting psychosocial outcome measures embedded within a precision medicine trial for childhood cancer
Source: Cancer Med. 2024 Jun 19;13(12):e7339. doi: 10.1002/cam4.7339 (PMC11187165; doi:10.1002/cam4.7339)
Supplement: Supplementary file 2 — Appendix S2. [file CAM4-13-e7339-s002.docx]

**Appendix 2. Study data collection and analysis, by research question**

|  |  | **Intake** | **T0 – Parent** | **T1 – Parent** | **T1b – Parent** | **PRISM database** | **PRISM-Impact records** |
| --- | --- | --- | --- | --- | --- | --- | --- |
|  | Parent demographics (e.g., age, gender) | **-** | **x** | **-** | **-** | **x** | **-** |
|  | Child demographics and clinical data (e.g., age at diagnosis) | **-** | **-** | **-** | **-** | **x** | **-** |
| RQ1 | PRISM-Impact family and adolescent response rates | **-** | **-** | **-** | **-** | **-** | **x** |
|  | PRISM-Impact family and adolescent participation rates | **-** | **-** | **-** | **-** | **-** | **x** |
|  | PRISM-Impact attrition rates | **-** | **-** | **-** | **-** | **-** | **x** |
|  | Reasons for attrition | **-** | **-** | **-** | **-** | **-** | **x** |
|  | Preference for questionnaire format (online/paper) | **x** | **-** | **-** | **-** | **-** | **-** |
|  | Number of follow-up calls conducted to receive T0 | **-** | **-** | **-** | **-** | **-** | **x** |
|  | Number of follow-up calls conducted to receive T1 | **-** | **-** | **-** | **-** | **-** | **x** |
|  | Time from questionnaire being sent to being returned | **-** | **-** | **-** | **-** | **-** | **x** |
|  | Emotion Thermometer Tool – Domains: distress, anxiety, depression and anger, and need for help | **x**^b^ | **x** | **x** | **x** | **-** | **-** |
|  | Indications of distress | **x** | **x** | **x** | **x** | **-** | **-** |
|  | Time taken for psychologist to follow-up on indications of distress | **-** | **-** | **-** | **-** | **-** | **x** |
|  | Number of participants that the study psychologist deemed to be in immediate risk of harm upon follow-up | **-** | **-** | **-** | **-** | **-** | **x** |
| RQ 2 | Reason for consenting to PRISM-Impact (open-ended) ^a^ | **-** | **x** | **-** | **-** | **-** | **-** |
|  |  |  |  |  |  |  |  |
|  |  |  |  |  |  |  |  |
|  |  |  |  |  |  |  |  |
|  |  |  |  |  |  |  |  |
|  |  |  |  |  |  |  |  |
|  |  |  |  |  |  |  |  |
|  |  |  |  |  |  |  |  |
|  |  |  |  |  |  |  |  |
| RQ 4 | Rating of burden and benefit of being involved in PRISM-Impact (1=‘not at all’ to 5=‘very much) ^a^ | **-** | **x** | **x** | **x** | **-** | **-** |
|  | Whether participating in PRISM-Impact influenced their eagerness for their child to participate in PRISM (‘more eager’, ‘less eager’, ‘no effect) ^a^ | **-** | **x** | **x** | **-** | **-** | **-** |

*Note. RQ=Research question; T0=baseline; T1=post-return of PRISM results*

^a^ Adolescents were not asked these items in attempts to minimise their participation burden.

^b^ Only ‘distress’ domain of Emotion Thermometer Tool administered
