# Supplementary material for: The feasibility and acceptability of collecting psychosocial outcome measures embedded within a precision medicine trial for childhood cancer
Source: Cancer Med. 2024 Jun 19;13(12):e7339. doi: 10.1002/cam4.7339 (PMC11187165; doi:10.1002/cam4.7339)
Supplement: Supplementary file 3 — Appendix S3. [file CAM4-13-e7339-s004.docx]

**Appendix 3. Formulas for response, participation, and attrition rates**

|  | **Response rate** | **Participation rate** | **Attrition rate** |
| --- | --- | --- | --- |
| **Family*** | Number of eligible families who consented to participate in PRISM-Impact  ÷  Number of eligible families enrolled on PRISM eligible for PRISM-Impact  ×  100% | Number of eligible, consenting families who had at least one family member complete T0  ÷  Number of eligible families who consented to participate in PRISM-Impact and remained eligible at time T0 was sent  ×  100% | **-** |
| **Parent** | **-** | **-** | [(Number of eligible parents who returned T0 – number of eligible parents who returned T1)  ÷  Number of eligible parents who returned T0  ×  100% |
| **Adolescent** | Number of eligible adolescents whose parents consented for them to participate in PRISM-Impact  ÷  Number of eligible families in PRISM with a child aged 12-17 years at enrolment and eligible for PRISM-Impact  ×  100% | Number of eligible, consenting adolescents who completed T0  ÷  Number of eligible adolescents who consented to participate in PRISM-Impact and remained eligible at time T0 was sent  ×  100% | (Number of eligible adolescents who returned T0 – Number of eligible adolescents who returned T1)  ÷  Number of eligible adolescents who returned T0  ×  100% |

**We calculated response and participation rates per family as it was not possible to determine the number of eligible parents per family; We excluded families/participants whose response or participation status was pending (i.e., follow-up was ongoing and the maximum number of follow-up calls had not been reached or if they were still awaiting their PRISM results) from these calculations.*
