# Supplementary material for: The feasibility and acceptability of collecting psychosocial outcome measures embedded within a precision medicine trial for childhood cancer
Source: Cancer Med. 2024 Jun 19;13(12):e7339. doi: 10.1002/cam4.7339 (PMC11187165; doi:10.1002/cam4.7339)
Supplement: Supplementary file 4 — Appendix S4. [file CAM4-13-e7339-s003.docx]

**Appendix 4. Factors used in the multivariable logistic regression models**

| **Model** | **Variables tested** |
| --- | --- |
| Parents decision whether to participate in PRISM-Impact | - child’s baseline relapse status - child’s age - treating hospital - child’s diagnosis (liquid or solid) |
| Parents’ attrition between T0 and T1* | - parent age - parent gender - child’s diagnosis (liquid or solid) - child’s baseline relapse status - whether or not the family received treatment recommendation/s - rating of PRISM-Impact benefit at T0 - rating of PRISM-Impact burden at T0 - Emotion Thermometer Tool (ETT) scores at T0 |

**(excluding parents with missing data on tested covariates, parents who have yet to receive T1, parents who had a ‘pending’ status on T1, and bereaved parents)*
